# Defects That Magnetize Beyond Monolayer PtSe_2_

**DOI:** 10.1002/smll.73946

**Published:** 2026-05-24

**Authors:** Ilias M. Oikonomou, Danielle Douglas‐Henry, Mohammadreza Daqiqshirazi, Iva Plutnarová, Zdeněk Sofer, Thomas Brumme, Valeria Nicolosi, Thomas Heine

**Affiliations:** ^1^ Faculty of Chemistry and Food Chemistry Dresden University of Technology Dresden Germany; ^2^ Centre for Research on Adaptive Nanostructures and Nanodevices (CRANN) Centre for Advanced Materials and BioEngineering Research (AMBER) Trinity College Dublin Dublin Ireland; ^3^ School of Chemistry Trinity College Dublin Dublin Ireland; ^4^ Center for Advanced Systems Understanding (CASUS) HZDR Görlitz Germany; ^5^ Department of Inorganic Chemistry University of Chemistry and Technology Prague Prague Czech Republic; ^6^ Department of Chemistry and ibs for Nanomedicine Yonsei University Seoul Republic of Korea

**Keywords:** aberration‐corrected STEM, defects, density functional theory, magnetism, multilayer, PtSe_2_, two‐dimensional

## Abstract

Defect‐induced magnetism in two‐dimensional (2D) materials holds enormous potential for next‐generation spintronic and quantum devices, yet its realization beyond monolayer remains elusive. In this work, we investigate the emergence and modulation of magnetism in multilayer PtSe_2_ via a combined theoretical–experimental approach, integrating hybrid density functional theory (DFT) calculations with aberration‐corrected scanning transmission electron microscopy (AC‐STEM). In multilayer PtSe_2_ with the presence of Pt vacancies, magnetism is typically quenched due to interlayer interactions, but it can be restored by complex defect structures comprising, in addition to a Pt vacancy, a Pt_Se_ antisite. These configurations induce magnetic moments of up to 3.16 µ*_B_* and give rise to a two‐dimensional half‐metallic state in bilayer PtSe_2_, which is highly desirable for spin‐polarized transport. Furthermore, the electronic and magnetic properties can be tuned by nearby Se vacancies, which drive transitions between different types of magnetic states. When embedded in the middle layer of trilayer PtSe_2_, this combination of defects extends the magnetic moments beyond the defect‐carrying layer. Overall, these findings demonstrate that defect engineering enables robust magnetic phase control and spin‐filtering behavior without external doping or strain, establishing PtSe_2_ as a tunable 2D magnetic material platform for scalable, room‐temperature spintronic and valleytronic applications.

## Graphical abstract

The presence of a complex point defect consisting of a Pt vacancy (black circle) and a Pt_Se_ antisite (red circle) revives and increases the quenched magnetism in bilayer PtSe_2_. Magnetic properties can be further modulated by the presence of an Se vacancy (blue circle), which can convert the ferromagnetic behavior into antiferromagnetic when it occurs neighboring the Pt vacancy.

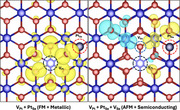

## 1 Introduction

Noble metal dichalcogenides are a subcategory of transition metal dichalcogenides (TMDs) in which the metal belongs to group 10 of the periodic table (M = Pt, Pd) [1]. Their bulk morphology has been known for many years, but their layered morphology was only proposed by Miró et al. in 2014 [2]. Among noble metal dichalcogenides, PtSe_2_ is particularly notable for its electronic tunability. First‐principles calculations predicted the layer‐dependent bandgap of layered PtSe_2_, where the monolayer exhibits an indirect bandgap and, after a few layers, transforms into a semimetal [2, 3, 4, 5, 6]. These predictions were confirmed by the first successful synthesis of monolayer PtSe_2_ by Wang et al. [7]. The electronic structure of PtSe_2_ can be tuned not only by varying the number of layers, but also by exploiting different stacking sequences that are accessible at elevated temperatures. Kempt et al. elucidated the impact of the stacking polymorphism on the electromechanical properties [8] while Ahn et al. recently proposed a layer‐dependent metal‐to‐insulator transition in different PtSe_2_ polymorphs in the presence of Se vacancies [9]. This tunable electronic structure, combined with high electron mobility and air stability, has established PtSe_2_ as a multi‐purpose material with a broad range of applications [10, 11, 12, 13]. However, to gain deeper insight into the potential applications of this material, it is essential to explore how the electronic structure is affected by point defects that can arise during the exfoliation or fabrication process.

Point defects strongly impact the properties and performance of 2D materials, as their presence can induce new physicochemical properties [14, 15]. In PtSe_2_, intrinsic point defects (e.g., vacancies) can induce 2D magnetism without the need for metallic doping via intercalation [16, 17]. Gao et al. investigated the role of individual Pt vacancies in inducing strong polarization in monolayer PtSe_2_ [18], while Zhang et al. proposed that an Se vacancy can generate magnetism only in the presence of strain [19]. The first experimental confirmation was reported by Avsar et al., who showed that the presence of Pt vacancies induces magnetism in metallic PtSe_2_ [20]. Later, the same team revealed magnetic transitions in mono‐ and bilayer PtSe_2_, where the former is antiferromagnetic, and the latter is ferromagnetic [21]. The magnetism originates from the 4p orbitals of the Se atoms surrounding the Pt vacancy [22]. The net magnetization depends on several factors (strain, defect density, layer thickness) [22], with the Kondo effect also being present [23]. Metallic edges have also been shown to be responsible for the occurrence of magnetism [24]. However, most previous studies have focused on monolayer systems, neglecting the influence of interlayer interactions. Furthermore, due to computational cost, studies on bi‐ and trilayer systems have been limited to higher defect densities, and complex defect configurations have so far been ignored.

In this work, we refine the structure–property interplay governing induced magnetism in layered PtSe_2_. We combine DFT calculations with AC‐STEM imaging to elucidate the quenching of magnetism relative to the monolayer and to uncover how complex defect configurations drive the emergence of novel electronic and magnetic properties. Initially, we investigate individual Pt vacancies in bi‐ and trilayer structures, considering different defect densities and defect positions. However, defects tend to aggregate into complex configurations, which are usually overlooked because of their high complexity and demanding computational requirements. Low‐voltage AC‐STEM imaging of liquid‐phase exfoliated PtSe_2_ is crucial for distinguishing such complex defects across different flakes. Liquid‐phase exfoliation (LPE) enables the scalable production of few‐layer 2D inks and facilitates the identification of dominant defect configurations within a single batch [25, 26]. Complex defects comprising a Pt vacancy and a Pt_Se_ antisite can substantially modify the electronic structure of multilayered structures and can even reverse the quenched magnetism arising from interlayer interactions between successive layers. We also consider the possibility that this complex defect extends magnetic moments beyond the defective layer in a trilayer structure. Additionally, we elucidate the effect of Se vacancies in combination with Pt vacancy and Pt_Se_ antisite as a means to tune 2D magnetism. Overall, our research demonstrates the pronounced impact of complex defects on tailoring the electronic structure and enabling 2D magnetism in layered PtSe_2_ beyond the monolayer.

## 2 Results

### 2.1 Magnetism and Electronic Transitions From Mono‐ to Bilayer PtSe_2_

Several studies revealed the presence of defect‐induced magnetism in monolayer PtSe_2_, with magnetic centers typically localized around Pt vacancies [18, 21, 22]. We confirm these results computationally: In a 5 × 5 supercell, which is our choice for single‐site defects throughout this work, the total magnetic moment amounts to 4 µ*_B_*. The calculations further show that the Se atoms are symmetrically distributed around the Pt vacancy with a constant separation of 5.19 Å, whereas in the pristine monolayer their separation is 5.04 Å (Figure S1a). The defect states are localized in the middle of the energy bandgap, which is therefore reduced to 0.48 eV (Figure S1b), compared to 1.68 eV in the pristine monolayer.

However, both Avsar et al. [21] and Manchanda et al. [22] reported a reduction in the total magnetization in bilayer PtSe_2_, with the latter also attributing this effect to the defect density. To clarify the origin of this quenching of magnetism, we investigate how a Pt vacancy modifies the local structural environment in the bilayer. We focus on two key structural parameters that may influence the reduction of magnetism: the interlayer distance and the positions of the Se atoms surrounding the Pt vacancy. Here, we define the interlayer distance as the average distance between the plane spanned by the Pt atoms in two consecutive layers. In the pristine structure, we find an interlayer distance of 5.17 Å (using PBE and MBD‐nl dispersion correction, see Methods), in agreement with the results published by Emrem et al. [27]. In contrast, for defective bilayers, we observe a reduction in the interlayer distance, which varies with defect density from 4.94 to 5.02 Å (Figure S2). Different defect densities are implicitly assessed through different supercell sizes and are defined in terms of the total number of missing atoms per layer of the corresponding element.

**FIGURE 1:**
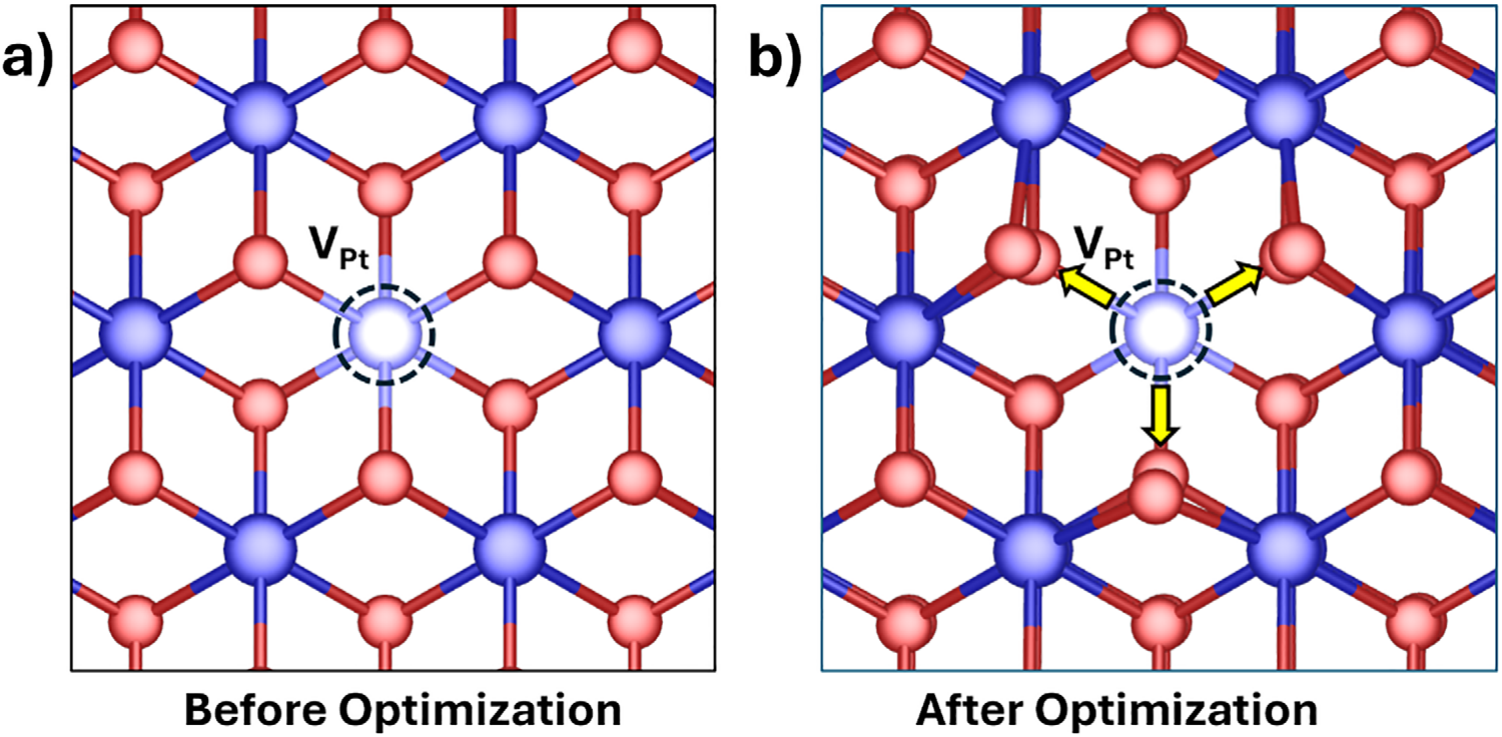
The effect of structural relaxation in bilayer PtSe_2_ with a Pt vacancy: (a) before and (b) after geometry optimization. The direction of atomic displacements is indicated by yellow arrows; Pt vacancies are marked with dashed black circles.

To understand the impact of the interlayer interactions on the magnetization, we created a Pt vacancy by removing one Pt atom from the unperturbed bilayer in the 5 × 5 supercell. The resulting total magnetic moment is essentially identical to that of the analogous monolayer PtSe_2_ with a Pt vacancy. When only the interlayer spacing is optimized while keeping all atomic positions fixed, the separation contracts to 5.02 Å and the net magnetization decreases to 3.26 µ*_B_*. Upon full relaxation, the Se atoms next to the vacancy shift toward the opposite layer, yielding an eclipsed Se stacking, as shown in Figure 1b. This relaxation increases the average distance between symmetry‑equivalent Se atoms across the bilayer to 5.36 Å. The combined effect of the reduced interlayer spacing and eclipsed stacking lowers the net magnetization of the bilayer to 2.00 µ*_B_*, compared with 4.00 µ*_B_* for the monolayer.

**FIGURE 2:**
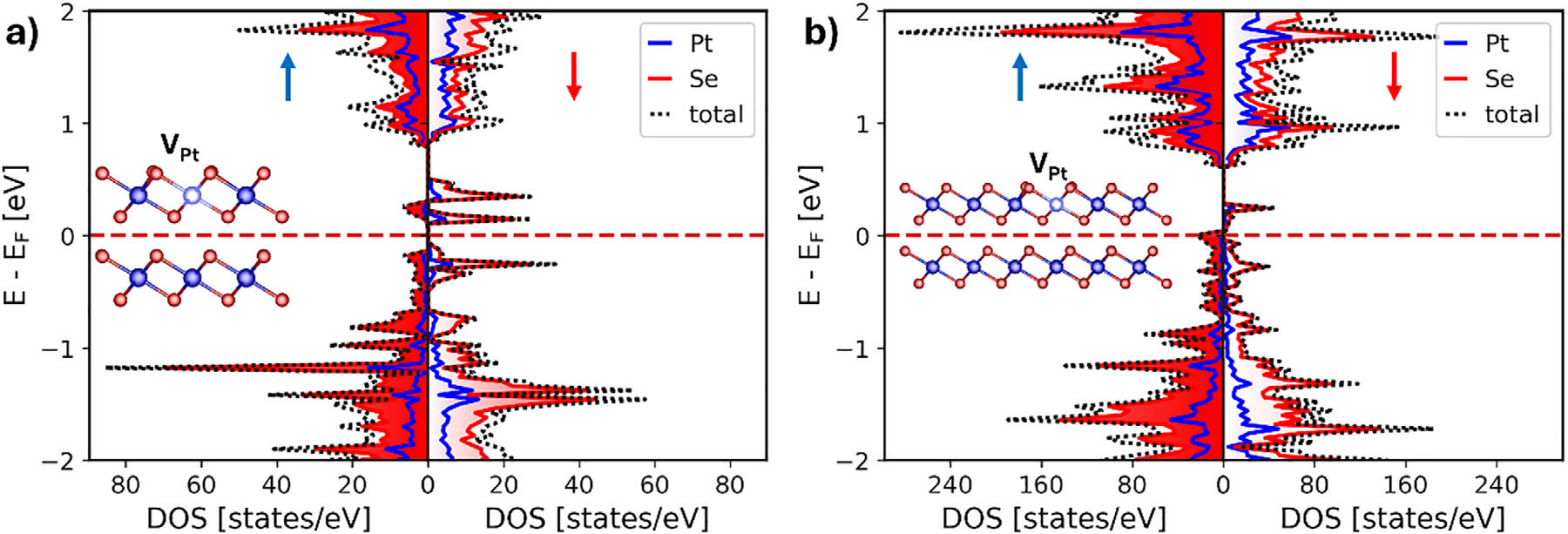
Spin‐polarized projected density of states (PDOS) for bilayer PtSe_2_ with a Pt vacancy in (left) a 3 × 3 and (right) a 6 × 6 supercell. Spin‐up and spin‐down channels are indicated by blue and red arrows, respectively. Note that the band structure refers to a single point defect per unit cell.

Individual Pt vacancies give rise to a finite magnetic moment: in the 3 × 3 supercell, the high‑spin state is energetically favored over the diamagnetic state by 0.31 eV, whereas in the 6 × 6 supercell this energy difference is reduced to 80.2 meV. The system with an 11.1% defect density remains semiconducting with an indirect bandgap of 0.16 eV, and the vacancy‑induced defect states are localized near the Fermi level (Figure 2a). At a lower defect density of 2.8%, the defect states hybridize with the valence bands, driving a semiconductor‑to‑metal transition (Figure 2b). These calculations show that an isolated Pt vacancy generates a total magnetic moment of about 2 µ*_B_*. Indicatively, DFT calculations in Avsar et al., indicated the presence of a total 1.33 µ*_B_* in the 4 × 4 supercell using the PBE functional [21].

In the case of three layers, the presence of different defect positions affects the occurrence of magnetism. For a 5 × 5 supercell, when the Pt vacancy is located in the outer layer, we observe spin‑polarized bands with a total magnetization of 1.6 µ*_B_*. This is accompanied by local eclipsed stacking, as also observed for the bilayer. The system is a 2D half‐metal, where one spin channel is metallic while the other one is semiconducting (Figure S3a). In contrast, when the defect is located in the inner layer, the magnetic state is disfavored by 0.58 eV, and the ground state becomes diamagnetic (Figure S3b). These results are consistent with earlier studies showing that individual defects induce magnetism only when they are located in an outer layer [20, 22, 23]. The Pt vacancy in the inner layer triggers the semiconductor‐to‐metal transition. This layer selectivity raises an important question about the origin of magnetism in samples with three or more layers, since isolated Pt vacancies generate magnetic moments only in the outermost layers. Moreover, more complex defect configurations than single Pt vacancies can further modify the magnetic response, motivating the need for detailed structural characterization. Consequently, low‑voltage AC‑STEM measurements are crucial for resolving and identifying such complex defect types.

### 2.2 Low‐Voltage AC‐STEM Imaging

To prepare a variety of samples, PtSe_2_ powder was exfoliated using liquid‑phase exfoliation (LPE) [28, 29]. High‑resolution AC‑STEM imaging is well suited to characterizing complex point defects beyond single vacancies. Employing AC‐STEM gives us the benefit of Z‐contrast (atomic number contrast) imaging. This can be used for the atom‐by‐atom characterization and defect detection in the sample [30]. A low acceleration voltage of 60 kV is used to minimize knock‑on damage from the convergent STEM beam [31].

**FIGURE 3:**
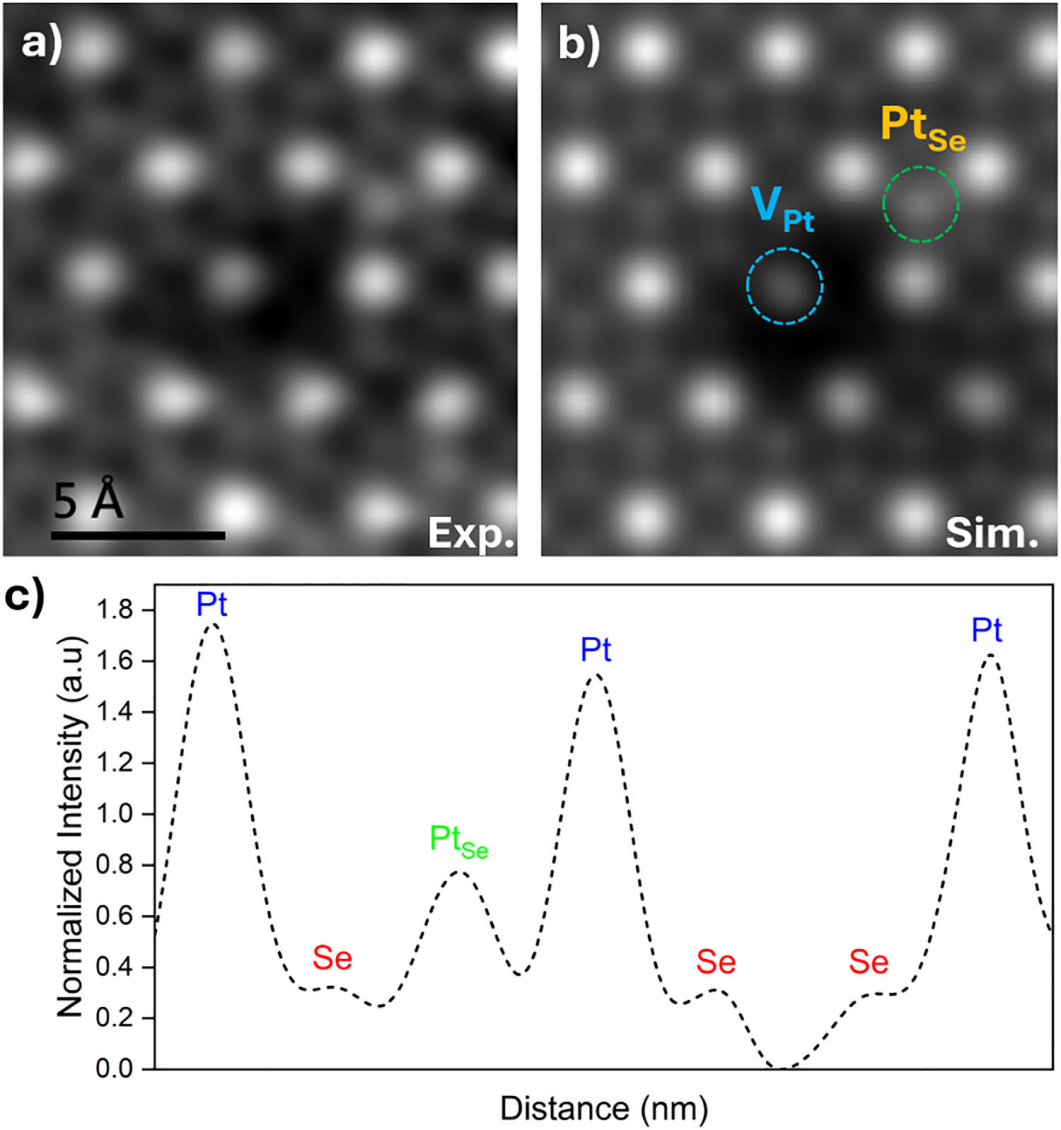
(a) Experimental and (b) simulated low‐voltage AC‐STEM image of liquid‐phase exfoliated PtSe_2_ illustrating a Pt vacancy (blue circle) and a Pt_Se_ antisite (green circle) surrounded by multiple Se vacancies. (c) Corresponding Z‐contrast line profile from experimental image confirming the presence of the Pt_Se_ antisite.

In Figure 3a, the octahedral 1T structure of PtSe_2_ is confirmed, but multiple point defects are also observed, most prominently multiple Se vacancies. To identify all defect species present in this region, multislice STEM imaging simulations [32] were performed using the DFT‐optimized geometry. By comparing the Z‑contrast between the experimental image (Figure 3a) and the simulated image (Figure 3b), a missing Pt atom is identified by its markedly reduced intensity relative to the surrounding Pt atoms. This missing Pt atom can migrate to the site of a neighboring Se atom, forming a Pt_Se_ antisite. According to our calculations, the possibility of forming a Pt interstitial between layers can be eliminated. Performing geometry optimization with the Pt atom located between the two layers, we observe the return to the initial crystal site. The presence of a Se vacancy next to the interstitial is also a metastable configuration that will lead to the return of Pt to the initial position. Analysis of the Z‑contrast line profile in the vertical direction confirms this hypothesis through an increased intensity at the Se position (Figure 3c), which is attributed to the presence of a Pt_Se_ antisite interrupting the Se sublattice. A similar Pt_Se_ antisite signature is also visible in AC‑STEM images reported by Ge et al. [23]. Measuring the defect density of Pt_Se_ antisites, in many different flakes, we result in an experimentally measured defect density of equal to 2.76  ± 0.3  ×  10^13^ *cm* ^−2^. A characteristic AC‐STEM image of the exfoliated sample is illustrated (Figure S4), where Pt vacancies and Pt_Se_ antisites are noted. The complex defect comprises a Pt vacancy and a Pt_Se_ antisite, with additional Se vacancies likely present but difficult to resolve unambiguously in multilayer structures. Defect generation can be attributed to the exfoliation process and/or electron‑beam irradiation from the convergent AC‑STEM beam. However, defect imaging at an operating voltage of 60 kV prevents the formation of Pt vacancies that could transform into Pt_Se_ antisites, as suggested by previously published works [33].

### 2.3 Electronic Structure of Complex Point Defects in Multilayered PtSe_2_

The complex defect discussed above, comprising a Pt vacancy, a Pt_Se_ antisite, and Se vacancies, is observed repeatedly in our LPE‑exfoliated samples. Minor variations are related to the number of Se vacancies. This defect is expected to influence the electronic and magnetic properties of few‐layer PtSe_2_, motivating dedicated first‐principles calculations. To unravel the interplay between different defect types, the initial calculations focus on the combined Pt vacancy–Pt_Se_ antisite complex, and Se vacancies are introduced in a subsequent step.

Because of the extended spatial range of the complex defect, we enlarged the computational model to a 6 × 6 supercell to suppress interactions between defect images in neighboring periodic images. A single point defect in a 6 × 6 supercell corresponds to 2.27 nm lateral spacing between the defects or to 2.25 ×  10^13^ *cm* ^−2^defect density. These values are very close to our experimental values, arising from the calculated defect density. Although the PtSe_2_ flake shown in Figure 3a is not a monolayer, we start our analysis from that to further understand how the Pt_Se_ antisite interacts with the Pt vacancy and modifies the electronic structure. As we observe in Figure S5a, the presence of the Pt atom in the Se crystal site does not massively affect the geometry, and only the Se atom adjacent to the antisite exhibits an increased distance to its symmetric counterpart. These defect states appear mid‑gap within the 0.6 eV bandgap and show pronounced spatial localization. The system carries a total magnetic moment of 4 µ*_B_*, demonstrating that an individual Pt_Se_ antisite does not generate additional magnetic moments (Figure S5b). Noteworthy, this complex defect in a single layer has a similar impact on the electronic and magnetic properties as an individual Pt vacancy.

In a bilayer PtSe_2_ with two point defects, it is not directly evident which layer hosts each defect. Electron‑channeling effects in AC‑STEM prevent an unambiguous assignment of the defects to individual layers. DFT simulations are thus used to explore the relative stability and evolution of defects among alternative configurations. Our calculations suggest that point defects are by 0.84 eV thermodynamically more stable if distributed in the same layer. The formation energy of different individual and complex defects for bilayer PtSe_2_ is summarized in Table S1.

**FIGURE 4:**
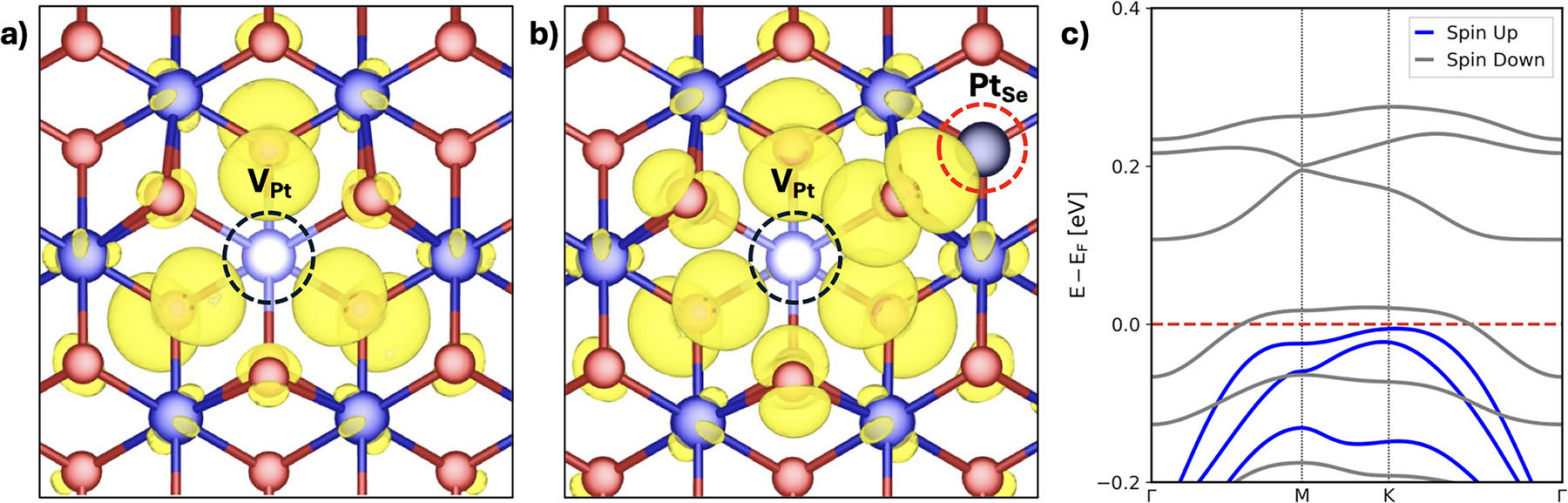
Spin densities of bilayer PtSe_2_ with (a) an isolated Pt vacancy, (b) a Pt vacancy and a Pt_Se_ antisite, and (c) the corresponding band structure. The supercell size in all cases was 6 × 6, and the isosurfaces are plotted with a 0.015 e/Å^3^ cutoff.

The presence of a Pt vacancy together with a nearby Pt_Se_ antisite in bilayer PtSe_2_ yields a magnetic moment of 3.16 µ*_B_*. This defect pair gives the highest calculated magnetic moment among all non‑monolayer configurations considered in this study. A further induction of complex defects offers an effective strategy to mitigate the magnetism quench in multilayer structures without relying on external doping. The enhancement of the total magnetic moment cannot be attributed solely to the Pt_Se_ antisite. As shown in the Supporting Information, an isolated Pt_Se_ antisite exhibits diamagnetic behavior with a bandgap of 0.7 eV, closely matching the pristine bilayer (Figure S6). In the spin‑density map (Figure 4b), the magnetic moments appear localized in the vicinity of the surrounding Se atoms. Hirshfeld analysis reveals that the combined Pt‐vacancy and Pt_Se_ defect induces an extended electronic perturbation with the Se atom adjacent to the Pt_Se_ antisite carrying a charge of −0.024e^−^ and a local moment of 0.52 µ*_B_*. This particular defect configuration in the bilayer gives rise to a two‑dimensional half‑metallic state. For this configuration, the spin‑up channel opens a 0.63 eV gap, while the spin‑down channel stays metallic (Figure 4c). The effect of spin–orbit coupling is negligible, as illustrated in Figure S7.

Most interestingly, our calculations indicate that this complex defect can also induce magnetism in trilayer PtSe_2_. For a defect in the outer layer, the Pt_Se_ antisite has two possible lattice sites available. If it occupies an outer Se site, geometry optimization shows that the Pt atom migrates back to its initial position, yielding a trilayer PtSe_2_ structure with a Se vacancy. Conversely, placing the antisite on an inner Se position produces a total magnetic moment of 2 µ*_B_* together with a narrow 25 meV bandgap (Figure S8a). Furthermore, when the defect complex lies in the central layer, the system becomes metallic (Figure S8c) with a total magnetization of 0.43 *µ_B,_* showing that point defects confined to an inner layer can generate magnetic moments in trilayer PtSe_2_ (Figure S8d). This configuration is energetically favored by 0.79 eV compared with the outer‑layer case and supports the view that complex defects underlie the emergence of magnetism in multilayer structures. The thermodynamic stability of this defect is proved by performing Climbing‐Image Nudged Elastic Band (CI‐NEB) calculations to identify the transition state between a structure having a Pt vacancy and a Pt_Se_ antisite in the inner layer and a structure with a Se vacancy. Our results indicate the presence of an energy barrier of approximately 1.5 eV, corresponding to a trapped configuration at 300K (Figure S9)

**FIGURE 5:**
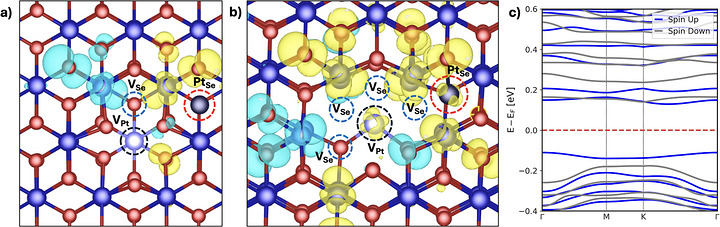
Spin densities of bilayer PtSe_2_ with (a) a Pt vacancy, a PtSe antisite, and one Se vacancy, (b) a Pt vacancy and a Pt_Se_ antisite and seven Se vacancies, and (c) the corresponding band structure. The supercell size in all cases was 6 × 6, and the isosurfaces are plotted with a 0.015 e/Å^3^ cutoff.

Se vacancies provide an additional handle to tune the magnetic behavior, even though earlier studies indicate that they do not by themselves induce magnetism in PtSe_2_ [19]. They are identified as the energetically most favorable point defect in this system [18], and their presence is also corroborated in our low‐voltage AC‐STEM imaging (Figure 3a). Coupling between a Se vacancy, the Pt vacancy, and the Pt_Se_ antisite is therefore a plausible defect configuration. For a Se vacancy located far from the defect core, the net magnetization rises to 2.47 µ*_B_*, exceeding the value obtained for an isolated Pt vacancy. Energetically, the configuration where the Se vacancy binds to the other defects is favored by 1.79 eV. This trend highlights the propensity of point defects to aggregate and form increasingly complex configurations. The total magnetization in this case is 0 µ*_B_*; nevertheless, Figure 5a shows local magnetic moments around the complex defect that couple antiferromagnetically and cancel each other. The band structure simultaneously changes from metallic to semiconducting with a gap of 0.37 eV, whereas all other defect types considered here retain a metallic character. When the concentration of Se vacancies is raised to match the AC‑STEM observations (Figure 3a), the system develops a net magnetization of 2 µ*_B_*. The spins surrounding the defects adopt non‑equivalent orientations (Figure 5b), resulting in an overall ferrimagnetic state. With several Se vacancies, the bandgap shrinks to 0.22 eV (Figure 5c), showing that defect configurations can switch magnetism between ferro‑, antiferro‑, and ferrimagnetic states and tune the electronic structure between metallic and semiconducting.

## 3 Conclusions

Through DFT calculations guided by AC‑STEM imaging, we identify the critical role of complex defect configurations in controlling the magnetic and electronic properties of multilayer PtSe_2_. Magnetism is strongly quenched when going from monolayers to bilayers, which is attributed to the reduced interlayer distance and the disruption of the eclipsed stacking caused by missing Pt vacancies. In contrast to previous published work [22], different defect densities produce comparable total magnetic moments but drive a transition from semiconducting to metallic behavior. Individual Pt vacancies in trilayers are position sensitive: they are diamagnetic when located in inner layers, but yield finite magnetic moments when they reside in the outer layer, which supports previous findings. Complex defect configurations, revealed by AC‑STEM as the prevalent defect state, are therefore essential for re‑establishing magnetism beyond the monolayer. Configurations that combine a Pt vacancy with a Pt_Se_ antisite generate the largest magnetic moments in bilayer PtSe_2_ while retaining a metallic character and avoiding any external doping. The magnetic state can be further tuned by Se vacancies: a single Se vacancy near the Pt vacancy–Pt_Se_ antisite complex stabilizes an antiferromagnetic configuration, whereas multiple nearby Se vacancies lead to a ferrimagnetic state. The presence of Se vacancies is responsible for a metallic‐to‐semiconducting transition consistent with previous experimental works [21], while the presence of a Pt vacancy has metallic character. These complex defects can also induce finite magnetic moments in trilayers, in contrast to the diamagnetic response of isolated Pt vacancies. This could explain the magnetic behavior in previously published works, since our calculation indicates the diamagnetic state as more stable. Overall, our results establish the mechanism underlying defect‑induced magnetism beyond monolayer PtSe_2_. Complex defects thus provide a versatile platform that acts as a lever to strongly tune PtSe_2_ without applying external fields or additional doping. More broadly, they underscore the importance of atomic‑level defect engineering in van der Waals materials for tailoring both magnetic order and electronic phase.

## 4 Methods

### 4.1 Synthesis of PtSe_2_ powder

Platinum sponge (Pt > 99.99%, Surepure Chemetals, USA) and selenium (Se > 99.9999%, granules 2–4 mm, Wuhan Tuocai Technology Co., Ltd., China) were placed in a quartz ampoule (25 × 100 mm) and melt sealed by an oxygen–hydrogen welding torch at a pressure of 1 mPa using an oil diffusion pump and liquid nitrogen cold trap. Selenium was used in 2 at.% excess. The ampoule was heated first to 1000°C using a heating rate of 1°C per minute. After 10 h at 1000°C, the ampoule was heated to 1280°C (heating rate 1°C per minute) for 30 min and then cooled to 1200°C using a cooling rate of 0.5°C per minute and subsequently cooled to room‐temperature at a cooling rate of 1°C per minute. The selenium excess condensed on the opposite side of the ampoule, and the formed crystalline block of PtSe_2_ was removed from the ampoule inside an argon‐filled glovebox.

### 4.2 Exfoliation of PtSe_2_

1 mg/mL PtSe_2_ powder was dispersed in isopropanol, followed by ultrasonication [34] for 6 h, under 40% amplitude with 6 s on and 2 s off. 30 min of high‐speed (3000 rpm) centrifugation was applied to separate the sediment from the supernatant of the fabricated ink.

### 4.3 Aberration‐Corrected STEM

The exfoliated flakes were transferred to Quantifoil grids for the AC‐STEM imaging. A low electron voltage of 60 kV was chosen to reduce the knock‐on damage to the specimen. High‐angle annular dark field (HAADF) STEM imaging was performed on the NION UltraSTEM, operated at 60 kV, with a 35 mrad probe convergence semi‐angle. All the images were analyzed using Gatan Microscopy Suite and averaged with SmartAlign [35]. Image processing included Smooth and Gaussian Blur filtering was applied to AC‐STEM images. Multislice STEM imaging simulations were performed with the abTEM code [36], incorporating also the PRISM algorithm [37].

### 4.4 DFT Calculations

1‐, 2‐, and 3‐layered 1T‐PtSe_2_ with individual Pt vacancies and complex defect structures have been fully optimized in 5 × 5 or 6 × 6 supercells (see text). The defect density is controlled by the supercell size. The distance between the periodically‐repeated defects along the z direction is at least 150 Å in all the cases (except for the trilayers with Pt_Se_ antisites and Pt vacancy, which was 50 Å) to avoid spurious superlattice effects imposed by the periodic boundary conditions. Pristine structures were calculated using primitive unit cells. The atomic positions of all the defective structures were optimized with FHI vibes [38] using either FHI‐aims as the solver or directly the internal optimizer of FHI‐aims. The Kohn‐Sham engine employed the PBE functional [39] augmented with MBD‐nl London dispersion correction [40], tight tier 1 numeric atomic orbital (NAO) in an 8 × 8 × 1 k‐point mesh grid (6 × 6 × 1 for 6 × 6 supercells). The force convergence threshold was set to 10^−2^ eV/ Å. Collinear spin polarization was included during structure optimization. Electronic structure calculations were performed with the FHI‐aims all‐electron code [41], using an intermediate tier 1 NAO with Heyd–Scuseria–Ernzerhof (HSE06) exchange‐correlation functional [42, 43, 44] accompanied by the MBD‐nl dispersion correction. HSE06 is an appropriate functional choice due to the localized nature of magnetic moments [45]. Post Self‐Consistent Field (SCF) Spin–orbit coupling [46] was employed in the case of a complex defect that included a Pt vacancy and a Pt_Se_ antisite on top of the HSE06 functional calculations.

## Conflicts of Interest

The authors declare no conflicts of interest.

## Supporting information




**Supporting File**: smll73946‐sup‐0001‐SuppMat.pdf.

## Data Availability

The data that support the findings of this study are openly available in Zenodo at https://dx.doi.org/10.5281/zenodo.17930674.
