# Supplementary material for: Defects That Magnetize Beyond Monolayer PtSe2
Source: Small. 2026 May 24;22(40):e73946. doi: 10.1002/smll.73946 (PMC13378613; doi:10.1002/smll.73946)
Supplement: Supplementary file 1 — Supporting File: smll73946‐sup‐0001‐SuppMat.pdf. [file SMLL-22-e73946-s001.pdf]

Defects that magnetize beyond monolayer $PtSe_2$

Ilias M. Oikonomou[1,2,3], Danielle Douglas-Henry[2,3], Mohammadreza Daqiqshirazi[1,5], Iva Plutnarová[4], Zdeněk Sofer[4], Thomas Brumme[1], Valeria Nicolosi[2,3] and Thomas Heine[1,5,6*]

[1] Faculty of Chemistry and Food Chemistry, Dresden University of Technology, 01069 Dresden, Germany

[2] Centre for Research on Adaptive Nanostructures and Nanodevices (CRANN), Centre for Advanced Materials and BioEngineering Research (AMBER) Centre, Trinity College Dublin, Dublin 2, Ireland

[3] School of Chemistry, Trinity College Dublin, Dublin 2, Ireland

[4] Department of Inorganic Chemistry, University of Chemistry and Technology Prague, Technicka 5, 166 28 Prague 6, Czech Republic

[5] Center for Advanced Systems Understanding (CASUS), HZDR, 02826 Görlitz, Germany

[6] Department of Chemistry and ibs for Nanomedicine, Yonsei University, Seoul 120-749, Republic of Korea

Supplementary Text

Individual Pt vacancy on monolayer $PtSe_2$

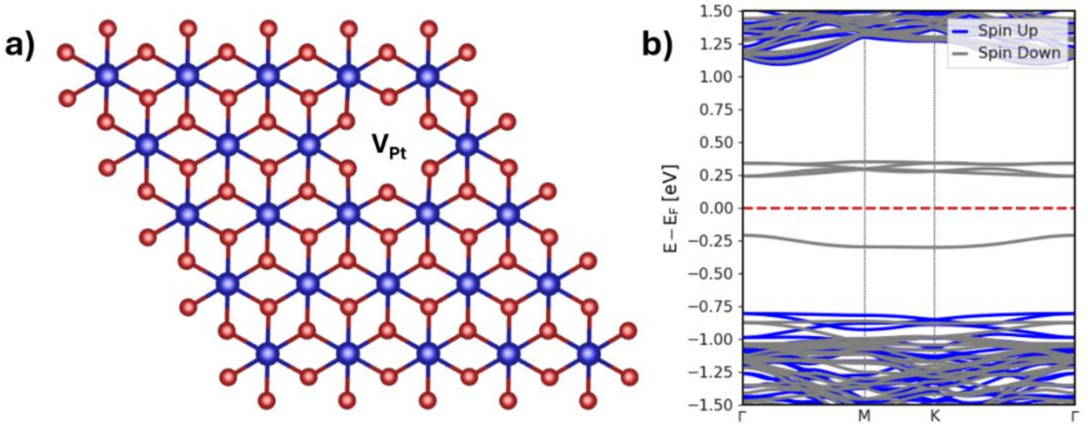

SI Figure 1. a) Geometry optimized monolayer $PtSe_2$ in a 5x5 supercell calculated with an individual Pt vacancy. The presence of a Pt vacancy increases the distance to the surrounding Se atoms to 5.19 Å from each other, instead of the 5.04 Å in the pristine structure. **b)** The corresponding band structure, with the total magnetization to be 4 μ_{B}, and the defect states are localized in the middle of the bandgap, which is reduced to 0.48 eV in comparison to 1.68 eV of the pristine monolayer.

Interlayer distance on different supercells in bilayer $PtSe_2$

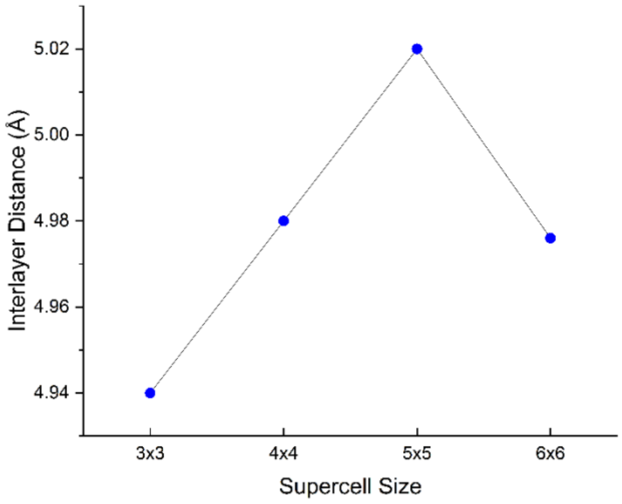

SI Figure 2. The effect of geometry optimization on the interlayer distance of bilayer $PtSe_2$ for different supercell sizes. In comparison to the pristine structure, which has an interlayer distance equal to 5.17 Å, the presence of an individual Pt vacancy significantly decreases it for different supercell sizes. We examine four different supercell sizes from 3x3 to 6x6, and we observe a reduction that ranges from 4.94Å to 5.02Å for odd numbers, while we observe a converged interlayer distance equal to 4.98Å for even numbers.

Individual Pt vacancies on tri-layer $PtSe_2$

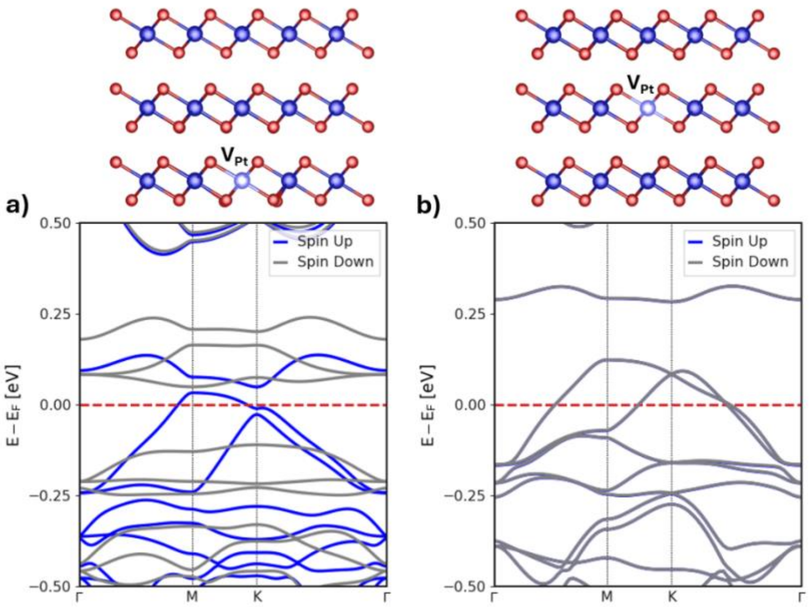

SI Figure 3. Atomistic model and band structures of tri-layer $PtSe_2$ with a Pt vacancy in **a)** the outer or **b)** the inner layer, in a 5x5 supercell. Different defect positions result in either ferromagnetic (outer) or diamagnetic (inner) behavior. Both defect positions result in metallic behavior, with the case of the outer layer being a 2D half-metal, with one spin channel being metallic and the other having an energy band gap equal to 0.16 eV. The corresponding Pt vacancy positions in each case are noted with light blue. Note that the band structure refers to a single point defect per unit cell.

Low-voltage aberration-corrected STEM imaging of liquid-exfoliated $PtSe_2$

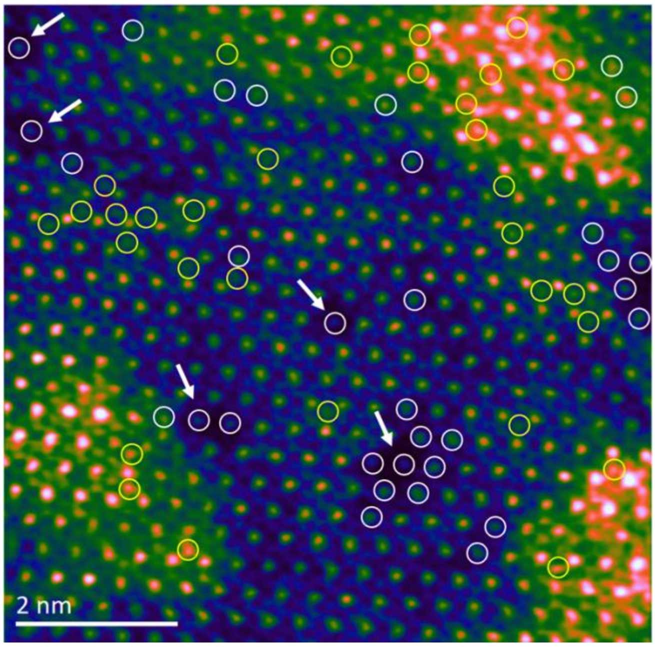

SI Figure 4. High-angle annular dark field (HAADF) STEM image of liquid-phase exfoliated $PtSe_2$. Pt_{Se} antisites are noted with yellow circles, while Pt vacancies are noted with white circles. Defect density of the two kinds of defects is approximately the same. In regions noted with white arrows, we observe an abrupt loss of the Z-contrast, implying the presence of multiple Pt vacancies along the perpendicular direction.

Pt_{Se} antisite and Pt vacancy on monolayer $PtSe_2$

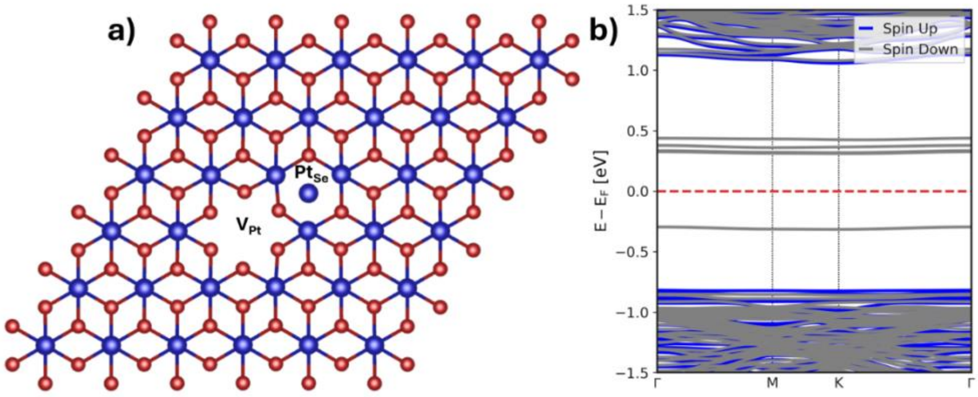

SI Figure 5. a) Geometry optimized monolayer $PtSe_2$ in a 6x6 supercell, including an individual Pt vacancy and a Pt_{Se} antisite, and **b)** the corresponding band structure. The presence of the Pt_{Se} antisite does not massively affect the local bonding environment, since only the position of the Se neighboring to the antisite changes. Defect states are localized in the middle of the band gap, which is equal to 0.6 eV. The presence of the antisite increases the bandgap in comparison to a case where only a Pt vacancy exists. The total magnetization remains the same and is equal to 4 μ_B.

Defect formation energy in bilayer $PtSe_2$

Case	Energy (eV)
Pt_{Se} antisite	1.23
Se vacancy	2.32
Pt vacancy	4.78
Pt vacancy + Pt_{Se} antisite	6.05
Pt vacancy + Pt_{Se} antisite + Se vacancy	6.56
Pt vacancy + Pt_{Se} antisite + 7x Se vacancies	20.18

SI Table 1: Defect formation energy of different individual and complex defects in a 6x6 supercell bilayer $PtSe_2$ using HSE06+MBD-nl.

Individual Pt_{Se} antisite on a bilayer $PtSe_2$

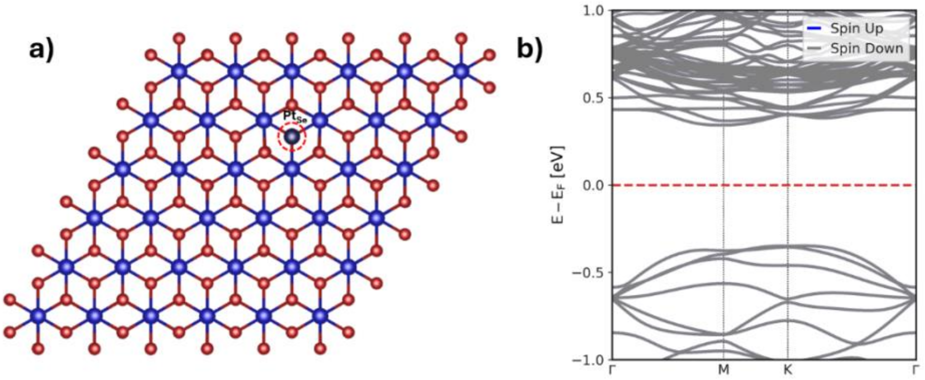

SI Figure 6. a) Atomistic model and **b)** corresponding band structure of a bilayer $PtSe_2$ with an individual Pt_{Se} antisite, in a 6x6 supercell. The energy band gap is 0.71, but since no spin-polarized bands are observed, the overall behavior is diamagnetic. Pt_{Se} antisite alone cannot induce magnetism in bilayer $PtSe_2$ as an individual Pt vacancy. Antisite atom is noted with black.

Spin-orbit coupling effect in bilayer $PtSe_2$, including a Pt vacancy and a Pt_{Se} antisite

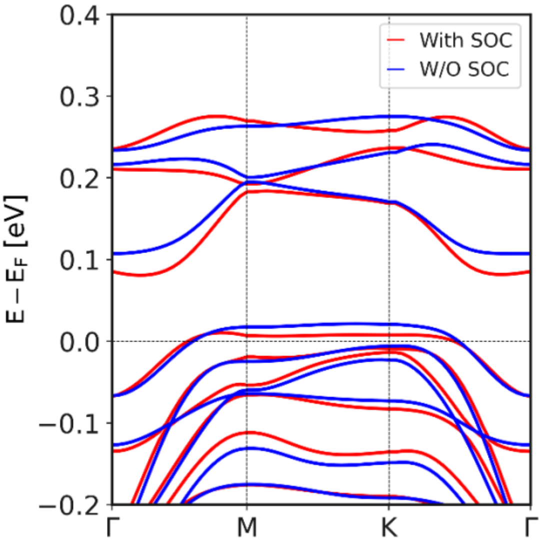

SI Figure 7. Electronic properties of bilayer $PtSe_2$, including an individual Pt vacancy and a Pt_{Se} antisite in a 6x6 supercell, including the effect of spin-orbit coupling. Band-splitting is almost negligible with respect to the electronic properties. The behavior of the system is metallic, with the total magnetization being 3.16 μ_B. The synergistic effect of the two defects is an indirect way to reverse the magnetism quench in the bilayer $PtSe_2$ and increase the total magnetic moments without the need for external doping.

Pt_{Se} antisite and Pt vacancy on tri-layer $PtSe_2$

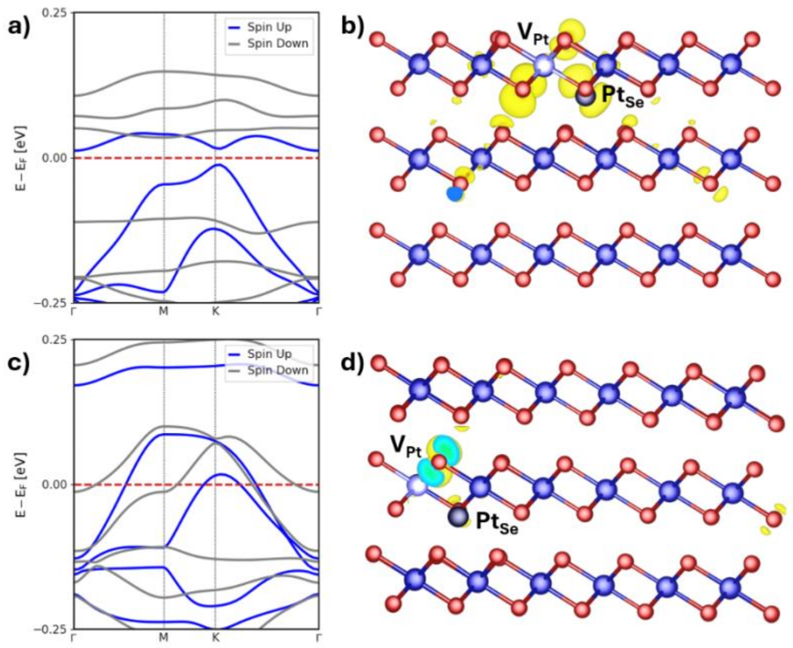

SI Figure 8. a), c) Electronic structure and **b), d)** plotted spin densities of tri-layer $PtSe_2$ with a Pt vacancy and a Pt_{Se} antisite in different positions, in a 6x6 supercell. When they occur in an outer layer, the structure exhibits semiconducting behavior with a narrow bandgap of 25 meV, while when they occur in the central layer, the structure becomes semiconducting, which is the most energetically stable position. Both cases induce the occurrence of magnetism. This is the first time we observe the magnetic

moments localized because of the presence of point defects in the central layer. The corresponding Pt vacancies in each case are noted with light blue, while the Pt_{Se} antisites with black. Note that the band structure refers to double point defect per unit cell. The isosurfaces are plotted using 0.015 e/Å³.

Thermodynamic stability of complex defects in trilayer $PtSe_2$

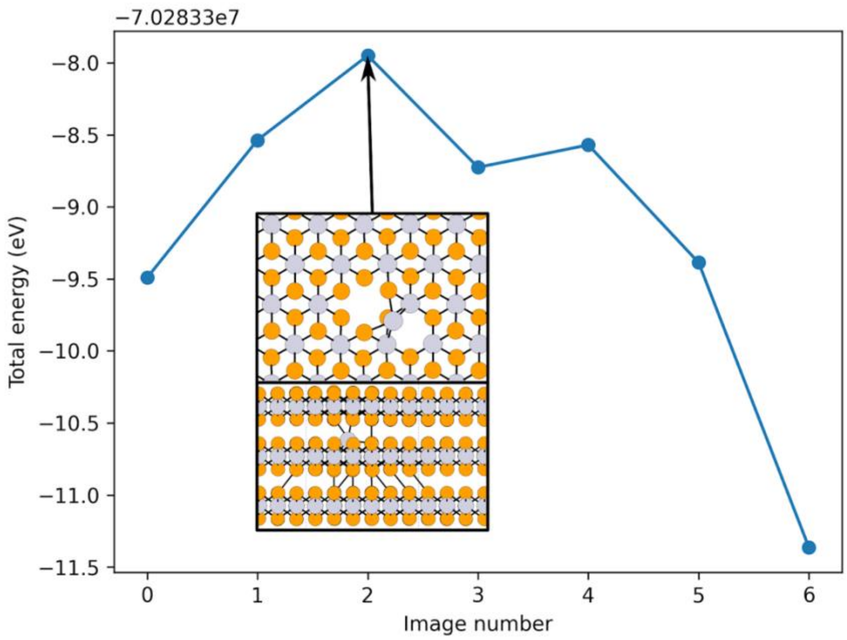

SI Figure 9 CI-NEB results for a transition from a system with Pt vacancy + Pt_{Se} antisite ("image 0") to a single Se vacancy ("image 6"). While the single Se vacancy is clearly lower in energy, the energy barrier of approximately 1.5 eV prevents the Pt of the Pt_{Se} vacancy from diffusing. This large barrier is due to the stronger interlayer interactions in $PtSe_2$. The insets show the structure of the transition state with the top view only showing the inner layer for clarity – grey - Pt; orange – Se
